# Supplementary material for: Microbiota composition in the lower respiratory tract is associated with severity in patients with acute respiratory distress by influenza
Source: Virol J. 2023 Feb 1;20:19. doi: 10.1186/s12985-023-01979-3 (PMC9891757; doi:10.1186/s12985-023-01979-3)
Supplement: Supplementary file 1 — Additional file 1. Supplementary Figure 1. Composition of the respiratory microbiota across all patients at phylum and genus level. A. Stacked barplot depicting the relative abundance at phylum level for all samples. B. Stacked barplot depicting the relative abundance at genus level for all samples. [file 12985_2023_1979_MOESM1_ESM.docx]

Supplementary material

​**Microbiota composition in the lower respiratory tract is associated with severity in patients with acute respiratory distress by influenza**

Alejandra Hernández-Terán^1,^ ^¶^, Angel E. Vega-Sánchez^2, ¶^, Fidencio Mejía-Nepomuceno^1^, Ricardo Serna-Muñoz^1^, Sebastián Rodríguez-Llamazares^1^, Iván Salido-Guadarrama^2^, Jose A. Romero-Espinoza^1^, Cristobal Guadarrama-Pérez^3^, JL Sandoval^4^, Fernando Campos^5^, Erika N. Mondragón-Rivero^1^, Alejandra Ramírez-Venegas^1^, Manuel Castillejos-López^6^, Norma A. Téllez-Navarrete^7^, Christopher E. Ormsby^1^, Rogelio Pérez-Padilla^1^, Joel A. Vázquez-Pérez^1*^.

**Supplementary Figure 1. Composition of the respiratory microbiota across all patients at phylum and genus level. A.** Stacked barplot depicting the relative abundance at phylum level for all samples. **B.** Stacked barplot depicting the relative abundance at genus level for all samples.
